# Supplementary material for: Attachment Styles as Protective and Amplifying Factors in Caregiver Psychological Distress: A Multicenter Cross-Sectional Study in Oncology and Chronic Disease Caregiving
Source: Healthcare (Basel). 2025 Oct 17;13(20):2612. doi: 10.3390/healthcare13202612 (PMC12563351; doi:10.3390/healthcare13202612)
Supplement: Supplementary file 1 [file healthcare-13-02612-s001.zip › healthcare-3854086-supplementary.pdf]

STROBE Checklist for Cross-Sectional Studies

		Recommendation	Location in Manuscript & Comments
**TITLE and ABSTRACT**			
	**1**	(a) Indicate the study's design with a commonly used term in the title or the abstract. b) Provide an informative and balanced summary of what was done and what was found.	(a) Title & Abstract: The design is clearly stated in both the title ("...A Multicenter Cross-Sectional Study") and the abstract ("This multicenter, cross-sectional study..."). (b) Abstract: The abstract is well-structured, covering Background/Objectives, Methods, Results, and a Conclusion. It provides a balanced summary of the study's key findings and implications
**INTRODUCTION**			
	**2**	Background/rationale: Explain the scientific background and rationale for the investigation being reported.	Introduction, Paragraphs 1-8: This section thoroughly establishes the context of caregiver burden in cancer and chronic illness, introduces attachment theory, and cites existing literature to build a clear rationale for investigating attachment styles as moderators of psychological distress.
	**3**	Objectives: State specific objectives, including any prespecified hypotheses.	Introduction, Final Paragraph: The objective is explicitly stated: "...our study methodically explores the pathways linking attachment styles with depression, stress, and anxiety, and their cumulative impact on caregiver burden." The aim to inform tailored support mechanisms serves as a clear hypothesis.
**METHODS**			
	**4**	Study design: Present key elements of study design early in the paper.	Section 2.1, Study Design and Participants, Paragraph 1: The first sentence of the Methods section clearly identifies the study design: "This multicenter cross-sectional study...".
	**5**	Setting: Describe the setting, locations, and relevant dates, including periods of recruitment, exposure, follow-up, and data collection.	Section 2.1 & 2.2: The setting is described as multiple hospitals across Turkey, specifically oncology clinics and the Tuzla State Hospital's home care clinic. The data collection period is clearly stated as "from October 2023 to September 2024."
	**6**	Participants: (a) Give the eligibility criteria, and the sources and methods of selection of participants.	Section 2.1, Study Design and Participants: This section provides detailed inclusion criteria (age, caregiver status, duration) and extensive exclusion criteria (psychiatric

		disorders, etc.) for both caregiver groups. The sources (clinics) and selection method (consecutive sampling of attendees) are clearly described.
**7**	Variables: Clearly define all outcomes, exposures, predictors, potential confounders, and effect modifiers. Give diagnostic criteria, if applicable.	Section 2.2, Data Collection: All primary variables are clearly defined by the validated scales used to measure them: Zarit Caregiver Burden Scale (outcome), DASS-21 (predictors/outcomes), and Relationship Scale Questionnaire (moderators/predictors). Criteria for patient conditions (e.g., stage 4 tumors) are also specified.
**8**	Data sources/measurement: For each variable of interest, give sources of data and details of methods of assessment (measurement). Describe comparability of assessment methods if there is more than one group.	Section 2.2, Data Collection: This section provides a detailed paragraph on each measurement tool (ZCBS, DASS-21, RSQ), including its purpose, scoring, and psychometric properties of the Turkish versions. Since the same tools were used for both groups, comparability is maintained.
**9**	Bias: Describe any efforts to address potential sources of bias.	Section 2.2 & 4.1: Bias is addressed by: 1. Using trained volunteer personnel who were "unaware of the patient's diagnosis" to administer surveys (Section 2.2). 2. Controlling for confounders in the multivariate regression analysis (Section 2.4). 3. A comprehensive discussion of potential biases (selection, self-report, cultural) in the dedicated Limitations section (4.1).
**10**	Study size: Explain how the study size was arrived at.	Section 2.2, Data Collection, Paragraph 5: The manuscript explains that the target of 500 participants per group was determined by a power analysis and that retrospective power analyses were also performed to confirm the final sample was adequately powered.
**11**	Quantitative variables: Explain how quantitative variables were handled in the analyses. If applicable, describe which groupings were chosen and why.	Section 2.4, Statistical Analysis: This section describes that normality was assessed via skewness/kurtosis and that variables were used in correlation and regression analyses. It also mentions the use of the Kruskal-Wallis test for comparing scores across categorized subgroups (e.g., cancer type).
**12**	Statistical methods: (a) Describe all statistical	(a) Section 2.4: Details descriptive statistics, normality tests, correlation analyses (Pearson),

		methods, including those used to control for confounding.<br>(b) Describe any methods used to examine subgroups and interactions.<br>(c) Explain how missing data were addressed.<br>(d) If applicable, describe analytical methods taking account of sampling strategy (eg, cluster sampling).<br>(e) Describe any sensitivity analyses.	and both univariate and multivariate linear regression to identify predictors and control for confounding.<br>(b) Section 2.4: Explicitly describes the use of the Hayes' PROCESS macro to test for moderation (interaction) effects.<br>(c) Section 2.4: This is clearly addressed: "The analysis was performed using a complete-case approach." "This approach led to the exclusion of 29 participants (17 cancer caregivers and 12 chronic disease caregivers) who did not complete all questionnaires."<br>(d) Not Applicable: The study used consecutive/convenience sampling, not a complex sampling design like cluster sampling.<br>(e) Section 2.2: Mentions a post-hoc power analysis was conducted to confirm the robustness of the findings.
**RESULTS**			
	**13**	Participants: (a) Report the numbers of individuals at each stage of the study—eg, numbers potentially eligible, examined for eligibility, confirmed eligible, included in the study, completing follow-up, and analysed.(b) Give reasons for non-participation at each stage. c) Consider use of a flow diagram.	Section 2.2, Paragraphs 3-4: The flow of participants is clearly described in the text. It provides the initial numbers approached and then lists the specific numbers excluded at each stage along with the reasons (e.g., age > 65, coexisting disorders, lack of consent), resulting in the final sample size for both groups. (c) A flow diagram is not used, but the textual description is sufficient and clear.
	**14**	Descriptive data: (a) Give characteristics of study participants (eg, demographic, clinical, social) and information on exposures and potential confounders. (b) Indicate the number of participants with missing data for each variable of interest.	(a) Table 1: "Sociodemographic and Clinical Features" provides a comprehensive summary of participant data, including age, gender, education, relationship to the patient, and cancer/disease types. (b) Section 2.4: The manuscript states a complete-case analysis was used, meaning there was no missing data in the final analyzed dataset.
	**15**	Outcome data: Report numbers of outcome events	Tables 2-6 & Figures 1-3: Summary measures (mean ± SD) are reported in Table 2.

		or summary measures.	Correlation coefficients are in Tables 3 & 4. Unstandardized (B) and standardized (β) coefficients from regression analyses are in Tables 5 & 6. Moderation effects are also numerically reported and visualized in Figures 1-3.
	**16**	Main results: (a) Give unadjusted estimates and, if applicable, confounder-adjusted estimates and their precision (eg, 95% confidence intervals). Make clear which confounders were adjusted for and why. (b) Report category boundaries when continuous variables were categorised. (c) If relevant, consider translating estimates of relative risk into absolute risk for a meaningful time period.	(a) Tables 5 & 6: The regression tables clearly present both "Univariate regression analysis" (unadjusted) and "Multivariate regression analysis" (adjusted) results, including coefficients (B), standard errors (SE), and confidence intervals. The confounders included in the multivariate models are clearly listed. (b) Table 5 Note: Category boundaries for some variables are defined, for example, "Type of Cancer (Non-common vs. common)". (c) Not Applicable: The study reports on associations and burden, not relative or absolute risk.
	**17**	Other analyses: Report other analyses done—eg, analyses of subgroups and interactions, and sensitivity analyses.	Results Section & Section 2.4: The results of the moderation (interaction) analyses using Hayes PROCESS macro are a central part of the results. Subgroup analyses comparing burden scores across different cancer types are also reported in the text (Paragraph 3 of the Results section).
**DISCUSSION**			
	**18**	Key results: Summarise key results with reference to the study objectives.	Section 4, Discussion, Paragraph 1: The discussion begins with a concise summary of the study's main findings, directly addressing the objectives by highlighting the differences between the two caregiver groups, the key predictors of burden, and the moderating role of attachment styles.
	**19**	Limitations: Discuss limitations of the study, taking into account sources of potential bias or imprecision. Discuss both direction and magnitude of	Section 4.1, Limitations: A detailed and self-critical subsection is dedicated to limitations. It thoroughly discusses issues such as cultural context, cross-sectional design (causality), selection bias from convenience sampling, heterogeneity of the chronic disease group,

		any potential bias.	self-report bias, and unmeasured confounders.
	**20**	Interpretation: Give a cautious overall interpretation of results considering objectives, limitations, multiplicity of analyses, results from similar studies, and other relevant evidence.	Section 4, Discussion: The entire discussion section provides a cautious interpretation. It compares findings to existing literature (citing numerous sources), explains the potential mechanisms behind the results (e.g., preoccupied attachment leading to over-involvement), and considers the study's limitations when drawing conclusions.
	**21**	Generalisability: Discuss the external validity (generalisability) of the study results.	Section 4.1, Limitations, Paragraph 1: Generalizability is explicitly discussed as a limitation, noting that the findings are "deeply rooted in this socio-cultural context [Turkey]" and that the specific sample (e.g., palliative cancer care, exclusion of caregivers >65) may not be representative of all caregivers.
**OTHER INFORMATION**			
	**22**	Funding: Give the source of funding and the role of the funders for the present study and, if applicable, for the original study on which the present article is based.	Funding Section: A dedicated section at the end of the manuscript clearly states: "This research received no external funding."

Source: von Elm E, Altman DG, Egger M, Pocock SJ, Gøtzsche PC, Vandenbroucke JP; STROBE Initiative. The Strengthening the Reporting of Observational Studies in Epidemiology (STROBE) statement: guidelines for reporting observational studies. PLoS Med. 2007 Oct 16;4(10):e296. doi: 10.1371/journal.pmed.0040296. PMID: 17941714; PMCID: PMC2020495.
